# Application of Secondary Electrospray Ionization Coupled with High-Resolution Mass Spectrometry in Chemical Characterization of Thermally Generated Aerosols

**DOI:** 10.1021/jasms.2c00222

**Published:** 2022-10-11

**Authors:** Tanja Zivkovic Semren, Shoaib Majeed, Maria Fatarova, Csaba Laszlo, Claudius Pak, Sandro Steiner, Guillermo Vidal-de-Miguel, Arkadiusz Kuczaj, Anatoly Mazurov, Manuel C. Peitsch, Nikolai V. Ivanov, Julia Hoeng, Philippe A. Guy

**Affiliations:** † PMI R&D, Philip Morris Products S.A., Quai Jeanrenaud 5, CH-2000 Neuchatel, Switzerland; ‡ Fossil Ion Technology, Promalaga, C. la Gitanilla, 17, 29004 Malaga, Spain

## Abstract

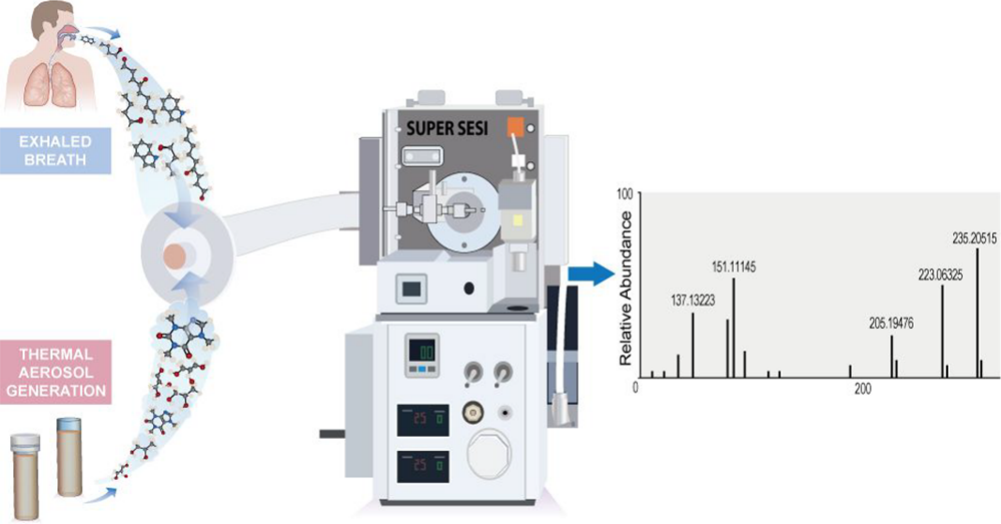

Inhalation as a route for administering drugs and dietary supplements has garnered significant attention over the past decade. We performed real-time analyses of aerosols using secondary electrospray ionization (SESI) technology interfaced with high-resolution mass spectrometry (HRMS), primarily developed for exhaled breath analysis with the goal to detect the main aerosol constituents. Several commercially available inhalation devices containing caffeine, melatonin, cannabidiol, and vitamin B12 were tested. Chemical characterization of the aerosols produced by these devices enabled detection of the main constituents and screening for potential contaminants, byproducts, and impurities in the aerosol. In addition, a programmable syringe pump was connected to the SESI–HRMS system to monitor aerosolized active pharmaceutical ingredients (APIs) such as chloroquine, hydroxychloroquine, and azithromycin. This setup allowed us to detect caffeine, melatonin, hydroxychloroquine, chloroquine, and cannabidiol in the produced aerosols. Azithromycin and vitamin B12 in the aerosols could not be detected; however, our instrument setup enabled the detection of vitamin B12 breakdown products that were generated during the aerosolization process. Positive control was realized by liquid chromatography-HRMS analyses. The compounds detected in the aerosol were confirmed by exact mass measurements of the protonated and/or deprotonated species, as well as their respective collision-induced dissociation tandem mass spectra. These results reveal the potential wide application of this technology for the real-time monitoring of aerosolized active pharmaceutical ingredients that can be administered through the inhalation route.

## Introduction

Drug delivery via inhalation has a rich history of more than 2000 years.^1^ It offers several advantages including a large absorptive surface area for efficient drug delivery, decreased drug metabolism, potentially fewer systemic side effects, and a relatively low drug concentration required to induce the desired physiological effects.^2^ The considerable potential of this drug delivery route has influenced the development and substantial production of devices^3,4^ that aerosolize the compound of interest for inhalation.^5,6^

Inhalation of drugs and dietary supplements has become increasingly popular, leading to the rapid development of modern inhalation technologies such as vaping devices that deliver specific compound supplements.^7^ This has prompted the need for advanced analytical techniques that can characterize the aerosols that such devices produce for detecting the main ingredients in the product and screening for potential contaminants, chemical impurities, and/or byproducts produced by the vaporization process.

Offline and online measurements are the two main approaches used to chemically characterize aerosols. The offline approach involves trapping of compounds on glass fiber filter pads or in impingers (single or multiple connected in series) prefilled with an appropriate solvent. The trapped compounds are then extracted and analyzed by hyphenated techniques such as gas (GC) or liquid (LC) chromatography coupled with mass spectrometry (MS) detection.^8−10^ The offline approach allows nontargeted screening, which enables the simultaneous detection of multiple compounds present in the aerosol and enriches less-abundant chemicals before analysis. However, the possibility of compound evaporation, potential discrepancies in the quantitative trapping of the entire chemical space in an aerosol, chemical cross-reactions that might occur during sample collection, and time-consuming procedures limit the offline approach.^11^

Online aerosol chemical characterization is mainly used as a targeted analytical technique. Its main advantage is its simple sampling procedure. However, the ionization process and commonly used detector methodology do not allow for comprehensive chemical monitoring. The development of new analytical techniques that allow rapid aerosol screening leads to progress in online chemical characterization. Current online methods for the chemical characterization of aerosols include direct analysis in real-time (DART) MS,^12^ Fourier-transform infrared spectroscopy (FTIR),^13^ selected-ion flow-tube (SIFT) MS,^14^ proton transfer reaction (PTR) MS,^15^ and single-photon ionization (SPI) MS.^16^ DART–MS, FTIR, SIFT–MS, PTR–MS, and SPI–MS can detect and semiquantify target compounds present in aerosols without sample handling. However, these techniques exhibit low resolution and selectivity in untargeted aerosol analyses. Hence, coupling these techniques with high-resolution (HR) MS could potentially improve the compound detection selectivity and enable the identification of chemicals using untargeted approaches. Recently, the atmospheric-pressure chemical ionization Orbitrap mass analyzer^17^ and extractive electrospray ionization–ultrahigh-resolution MS (EESI–Orbitrap)^18^ were introduced for the chemical speciation of environmental aerosols. Such techniques are of prime interest because they use high-resolution accurate mass detection, thereby improving the chemical identification and analysis of aerosol samples. The high sensitivity and selectivity of MS-based techniques combined with fragmentation information from tandem MS provide additional information on the chemical characterization of aerosols.

Secondary electrospray ionization (SESI) interface technology was recently introduced for the real-time monitoring of exhaled breath. SESI coupled with HRMS enables untargeted screening of exhaled breath samples, while high-resolution accurate mass tandem MS spectra can support chemical identification.^19−21^ This technique has also been applied to analyze aerosols generated from electronic cigarettes, confirming the potential of SESI for aerosol real-time analysis. The advantage of such an approach is that it mimics the real vaping process, which provides new insights into aerosol chemistry and avoids sample loss.^22^

The aims of the present study were to couple the SESI interface with HRMS for real-time analysis of exhaled breath to detect the main constituents of inhaled aerosols generated from vaping devices and to explore the applicability of SESI–HRMS for direct aerosol chemical characterization. Additionally, the SESI interface was modified to evaluate the aerosolization performance of drugs such as chloroquine (CQ), hydroxychloroquine (HCQ), and azithromycin.

## Experimental Section

### Chemicals and Test Items

Commercially available vaping devices with liquid formulations containing caffeine, melatonin, cannabidiol, and vitamin B12 were purchased from various suppliers. CQ and HCQ were synthesized by Wuxi (Hubei, China). Formic acid, deionized water, acetonitrile, ammonium acetate, ethanol, melatonin, caffeine, vitamin B12, azithromycin, 5,6-dimethylbenzimidazole, propylene glycol, and vegetable glycerin reference standards were purchased from Merck (Buchs, Switzerland). CQ, HCQ, and azithromycin were prepared for aerosolization by solubilizing the individual compounds in propylene glycol (5 mg/mL).

### Real-Time Exhaled Breath Analysis

**Figure 1:**
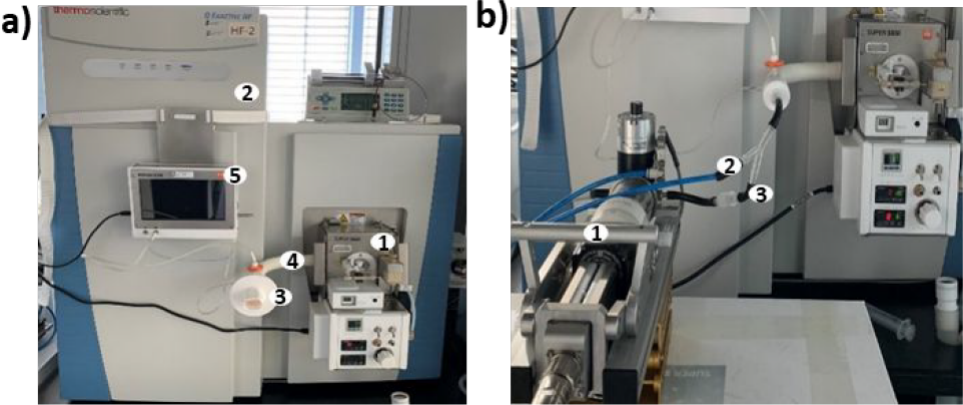
(a) Super secondary electrospray ionization (Super-SESI) (1), high-resolution mass spectrometry (HRMS) (2) instrument. In this configuration, the volunteer exhales directly through a disposable mouthpiece (3), which is connected directly to a sample line (4). The total exhaled flow, volume, and CO_2_ levels are monitored by the Exhalion system (5). (b) Programmable syringe pump (PSP) (1) used to generate an aerosol. The PSP is connected to the Super SESI–HRMS system for aerosol analysis. A nitrogen stream (2) is used to push the aerosol (3) through the Super SESI–HRMS detector. The vaping device is connected to the PSP system.

The exhaled breath of two healthy nonsmoker volunteers (female and male) was analyzed. Volunteers were informed of the aim of the study and verbally expressed their consent before participation. The study protocol was reviewed and approved by the internal review committee of Philip Morris International. The Super SESI source (Fossil Ion Technology, Madrid, Spain) was interfaced with an Orbitrap Q Exactive HF MS system (Thermo Fisher Scientific, Waltham, MA, USA) (Figure 1a). The subjects inhaled aerosols from commercially available vaping devices containing caffeine, melatonin, or vitamin B12, and then slowly exhaled through a disposable sterilized universal mouthpiece (C.D. Products S.A., Madrid, Spain) fitted into the Super SESI interface. The exhalation flow, volume, and CO_2_ levels were monitored using an Exhalion system (Fossil Ion Technology). Quality control of Exhalion system was performed every day before starting analysis with 5% CO_2_. The exhaled breath was delivered through a heated sampling tube (130 °C) to an ionization chamber (90 °C), where the compounds present in the exhaled breath were ionized with a stable electrospray (spray current 116 ± 3 nA) formed from 0.1% formic acid in water. The MS measurements were performed in positive and negative SESI modes at +3.6 and −3.4 kV, respectively, with a mass resolution setting of 240,000 (*m*/*z* 200) and a full-scan mass range of *m*/*z* 50–1400 (depending on the monitored compounds). Every day before starting the first exhaled breath analysis, quality control of the system was performed by analysis of standard gas containing α-terpinene (100 ppb) in positive ionization mode. SESI-HRMS calibration was performed every week with ambient air. The Xcalibur software package (version 4.2.47; Thermo Fisher Scientific) was used for data processing.

### Real-Time Analysis of Aerosols

Using commercially available vaping devices, aerosols were generated through the active breathing of the volunteer. Alternatively, to mimic the active breathing of the user, we connected a programmable syringe pump (PSP, Burghart Wedel, Germany) to a Super SESI instrument interfaced with a Q Exactive HF system (HRMS) to assess the aerosolization performance of the active pharmaceutical ingredient (API) (Figure 1b). Commercially available or in-house vaping devices were activated by inducing a volumetric flow of the generated aerosol (10–20 mL) within a 5-s duration (PSP setting parameters). Because of the backflush nitrogen stream present in the Super SESI interface (used for cleaning the inlet, improving the ionization efficiency, and preventing solvents and impurities from entering the MS vacuum system), another 1 L/min nitrogen stream was introduced into the setup to carry the aerosol into the SESI interface before MS detection. The same MS conditions and workflow were used for measurements as those used for exhaled breath analysis.

### Tandem MS

The analytes were chemically characterized by selecting the precursor ions of interest and analyzed using tandem MS. High-resolution accurate mass spectra were acquired using high-energy collision dissociation (HCD) conditions in positive and negative SESI modes under various collision energies, depending on the analyte of interest.

### Aerosol Trapping Experiments

The aerosol samples generated from the in-house or commercially available vaping devices were trapped on glass fiber filter pads with or without additional trapping in a microimpinger solvent. The two trapping methods were combined (filter pads and impinger) to avoid the loss of the compounds of interest in the case of compound partitioning between particulate matter and the gas vapor phase. The choice of solvent was compound-dependent; azithromycin, CQ, and HCQ aerosols were trapped in a microimpinger filled with 5 mL of ethanol, whereas caffeine, melatonin, vitamin B12, and 5,6-dimethylbenzimidazole were trapped in a microimpinger filled with 5 mL of water. The analytes were efficiently extracted from the glass fiber filter pads by adding 5 mL of the appropriate solvent to the filter pad and mixing for 30 min. The extracted analytes (5 mL) were combined with the solvent used in the microimpinger (total volume, 10 mL) and stored at +4 °C until analysis.

### Quantification of Aerosolized Compounds

The aerosolized and trapped compounds were quantified by the ultrahigh-pressure LC Vanquish Duo system (Thermo Fisher Scientific) coupled to a Q Exactive HF MS system (LC–HRMS). The column type, solvent, and gradient conditions used for each of the monitored compounds are reported in the Supporting Information (LC-HRMS conditions), as are details on the preparation of the calibration curves from the reference standards for quantifying the analytes present in the trapped aerosol samples.

## Results and Discussion

### Exhaled Breath Analysis After Inhalation of Aerosols Produced by Commercially Available Vaping Devices

As the Super SESI–HRMS was initially developed for analyzing exhaled breath, the first step was to monitor the chemicals present in the exhaled breath after a volunteer had inhaled the aerosol produced from the commercially available vaping devices. These devices were filled with an e-liquid containing a specific compound supplement, such as melatonin (for mitigating sleep disorders), caffeine (as a stimulant), or vitamin B12 (as an energy booster). The vaping devices were opened to extract the liquid, and the active ingredients (e.g., melatonin, caffeine, and vitamin B12) were quantified first to assess the information provided by the suppliers. Specific quantification methods were established, using LC–HRMS, as described in the Experimental section, and confirmed the presence and concentration of the expected active ingredient (Supporting Information, Table S1). Our main objective was to demonstrate the application of SESI technology for the chemical characterization of aerosols; therefore, the exact concentrations of the compounds in the aerosols were not the main focus considering that their levels could vary according to volunteer inhalation intensity.

Volunteers consumed the products by inhalation and exhaled the aerosol directly into the Super SESI–HRMS system. The Exhalion system connected to the Super SESI interface allowed us to collect a consistent volume of the exhaled breath. The flow rate, volume, and CO_2_ levels were recorded to ensure the reproducibility of the exhaled breath. Full-scan mass spectra were generated by scanning at *m*/*z* 50–1400 (depending on the target compound) in the positive or negative SESI acquisition mode. The goal was to confirm the presence of the active ingredient in the exhaled breath and detect other added compounds present in the e-liquid such as flavors and/or possible degradation products arising from the aerosolization process.

**Figure 2:**
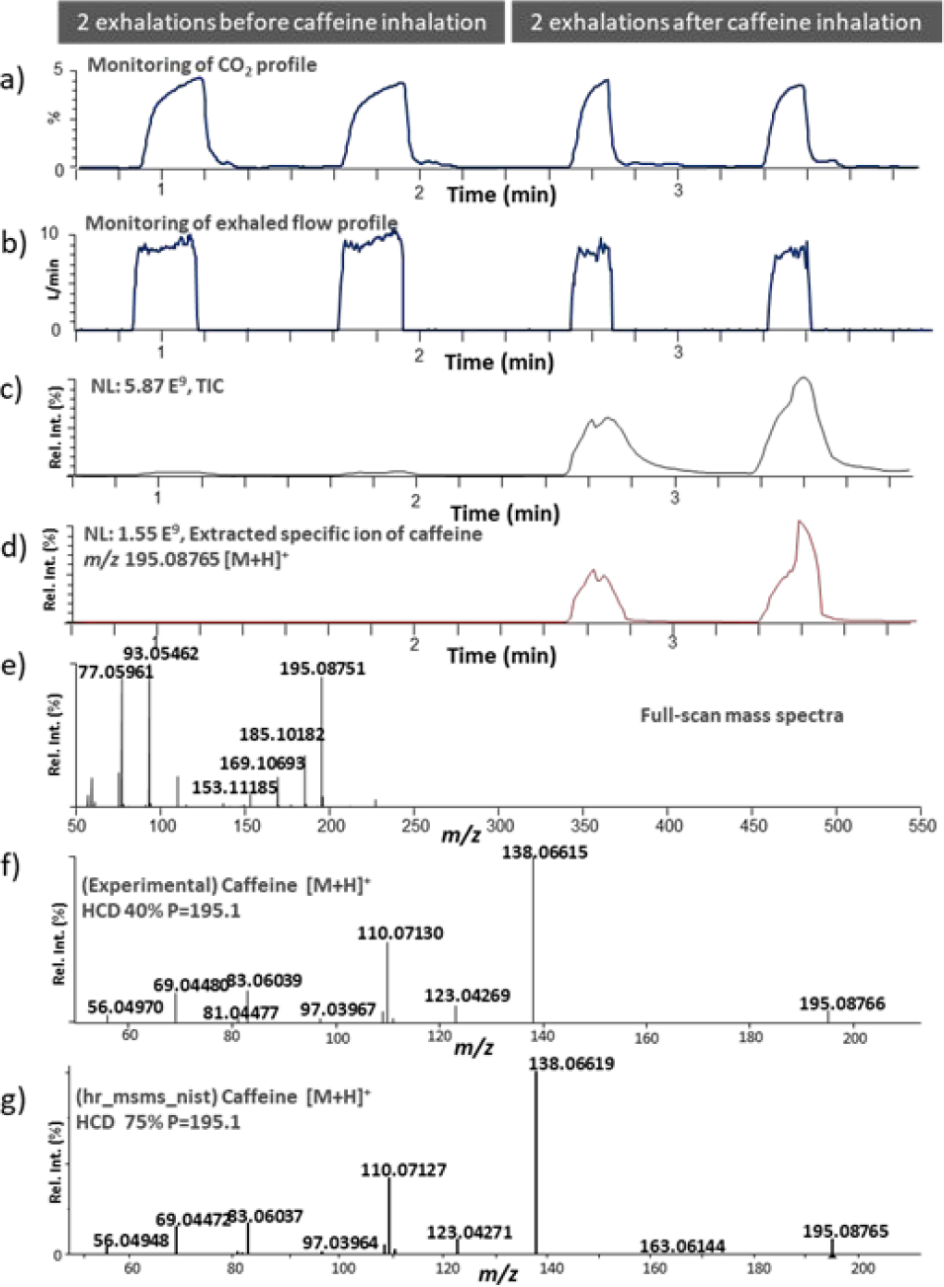
(a) Percentage CO_2_ levels and (b) exhaled breath volumetric flow (L/min) monitored by the Exhalion software. (c) Total ion current measured in two blank exhalations before and two exhalations after inhalation of aerosol from a caffeine-supplemented vaping device. (d) Extracted ion of *m*/*z* 195.08765 corresponding to the theoretical protonated molecular species of caffeine. (e) Subtracted full-scan secondary electrospray ionization (SESI) mass spectrum in the positive ionization mode (*m*/*z* 50–550). (f) High-energy collision dissociation (HCD) tandem MS spectrum from selecting *m*/*z* 195.1 at HCD 40 (SESI+). (g) HCD tandem MS spectrum of caffeine from the NIST20 library. rel int.: relative intensity.

Figure 2a,b shows the CO_2_ levels and exhaled volumes measured before and after use of a caffeine-supplemented vaping device. To standardize the exhaled breath measurements within and between the volunteers, the subjects were asked to exhale at a constant flow rate. The Exhalion system provides a visual clue that greatly facilitates this task. Monitoring both the exhaled volumetric flow and CO_2_ levels in real time allowed us to assess the reproducibility of the exhalation by the volunteers. The full-scan mass spectra generated in the positive SESI mode revealed the presence of the compounds that were detected as protonated species (Figure 2c–d). Caffeine was observed at *m*/*z* 195.08751, corresponding to the elemental composition of C_8_H_11_N_4_O_2_ with a mass accuracy of −0.78 ppm. In the full-scan mass spectrum, several other ions were also observed and putatively identified at *m*/*z* 77.05961 (propylene glycol, [M + H]^+^, −0.08 ppm), *m*/*z* 93.05462 (vegetable glycerin, [M + H]^+^, −0.22 ppm), *m*/*z* 153.11185 (propylene glycol, [2M + H]^+^, −1.89 ppm), *m*/*z* 169.10693 (C_5_H_11_N_7_, [M + H]^+^, −0.27 ppm), and *m*/*z* 185.10182 (vegetable glycerin, [2M + H]^+^, −0.76 ppm) (Figure 2e). The presence of the propylene glycol (*m*/*z* 153.11185) and vegetable glycerin (*m*/*z* 185.10182) dimers was mainly attributed to the high concentrations of these compounds in the e-liquid. The HCD tandem MS spectrum of the selected protonated ion of caffeine (*m*/*z* 195.1) corresponded well to that of the NIST20 library (Figure 2f–g). The aerosolization of caffeine was previously validated by Ueno et al.^23^ using mesh nebulizers; they confirmed that aerosolized caffeine inhaled by mice reached the brain and affected motor activity. Additionally, Yuan et al. showed that caffeine considered as a nonvolatile compound can be detected in exhaled breath by using an SPME-DART-MS approach after coffee intake.^24^

Similarly, the presence of melatonin was confirmed in the e-liquid from melatonin-supplemented vaping devices according to its reported concentration value. We also confirmed the effective aerosolization of melatonin from the exhaled breath of the volunteers (data not shown). Several studies have evaluated the effects of orally administering melatonin on mitigating sleep disorders in patients with asthma and chronic obstructive pulmonary disease and confirmed its benefits.^25,26^ Melatonin is also well-known for its beneficial effects on circadian rhythm regulation and cancer inhibition.^27^ Considering the significance of melatonin, its successful aerosolization could support research on melatonin administration via inhalation.

**Figure 3:**
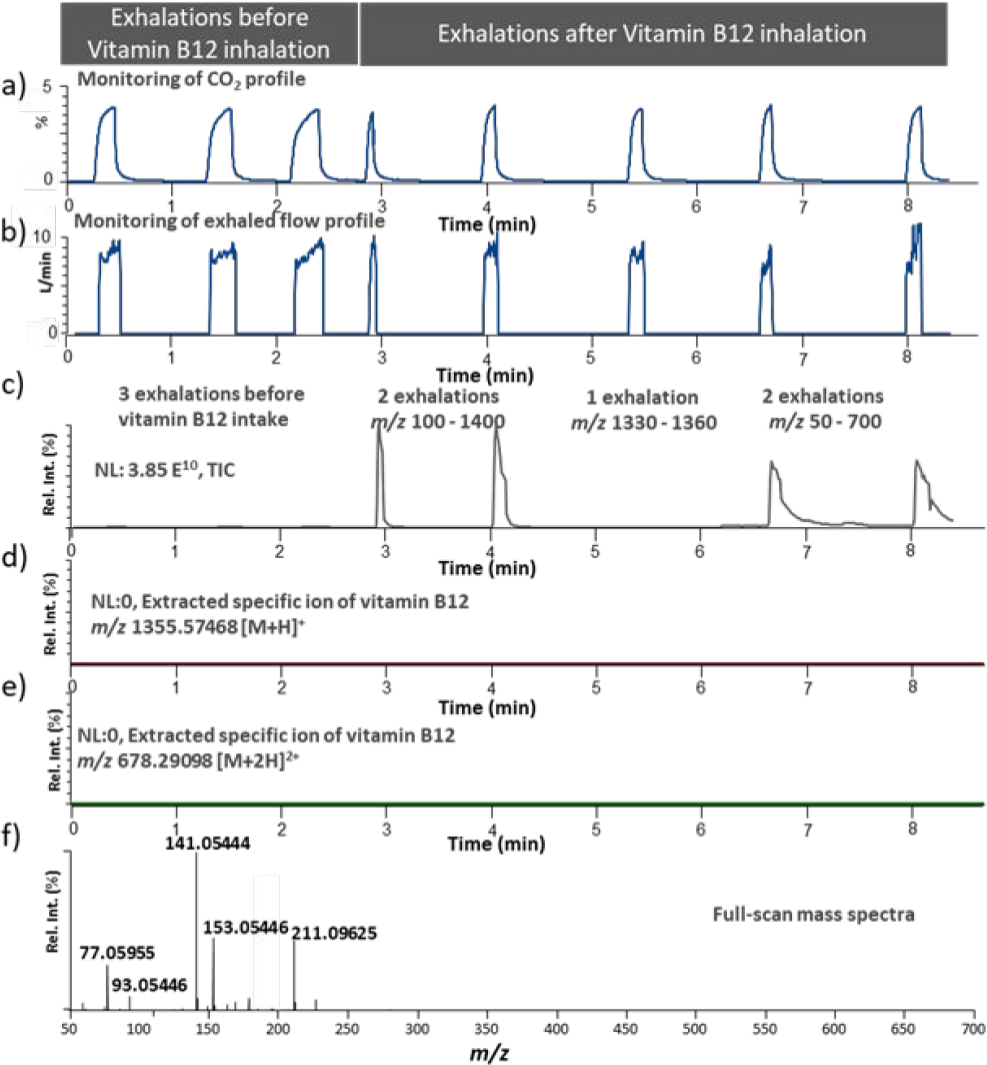
(a) Percentage CO_2_ levels and (b) exhaled breath volumetric flow (L/min) monitored by the Exhalion software. (c) Total ion current measured in the exhalation experiments with three exhalations before inhalation of aerosol from a vitamin B12-supplemented vaping device followed by two exhalations measured by scanning *m*/*z* 100–1400, one measured by scanning *m*/*z* 1330–1360, and the last two measured by scanning *m*/*z* 50–700 in the positive secondary electrospray ionization (SESI) mode. (d, e) Selected ion extractions (mass tolerance 5 ppm) of *m*/*z* 1355.57468 and *m*/*z* 678.29098 corresponding to the theoretical protonated molecular species of vitamin B12 (singly and doubly charged species, respectively). (f) Subtracted full-scan positive SESI mass spectrum generated by scanning *m*/*z* 50–700. rel int.: relative intensity.

For the vaping device that contained vitamin B12, the exhaled breath samples from a volunteer were analyzed in the SESI positive and negative full-scan acquisition modes by scanning the mass range of *m*/*z* 100–1400. However, selective extraction of singly or doubly charged ion species (*m*/*z* 1355.57468 and 678.29098, respectively) in the positive ionization mode did not confirm the presence of vitamin B12 (Figure 3a–f and Supporting Information, Figure S1, SESI negative mass spectrum). A narrower mass range (*m*/*z* 50–700) full-scan SESI mass spectra revealed the presence of alternative ions such as *m*/*z* 77.05955 (propylene glycol ([M + H]^+^, −0.08 ppm), *m*/*z* 93.05446 (vegetable glycerin ([M + H]^+^, −0.22 ppm), *m*/*z* 141.05444 (ethyl maltol ([M + H]^+^, −1.14 ppm), *m*/*z* 153.05446 (C_8_H_9_O_3_, −1.44 ppm), and *m*/*z* 211.09625 (C_11_H_15_O_4_, −1.83 ppm) (Figure 3f). In the SESI-negative ionization mode, the spectra revealed the presence of *m*/*z* 91.04008 (vegetable glycerin ([M + H]^−^, 0.36 ppm) and *m*/*z* 151.03990 (C_8_H_7_O_3_, −1.11 ppm) (Supporting Information, Figure S1).

A previous study investigated a vitamin B12 inhalation therapy in patients with pernicious anemia, in which vitamin B12 was delivered via a nebulizer and dust inhaler.^28^ In contrast to our results, they observed successful uptake via inhalation, which was confirmed by B12 activity in the urine and improved hematological parameters. Thus, we attempted to determine the cause of the absence of vitamin B12 in the exhaled breath in our study.

**Figure 4:**
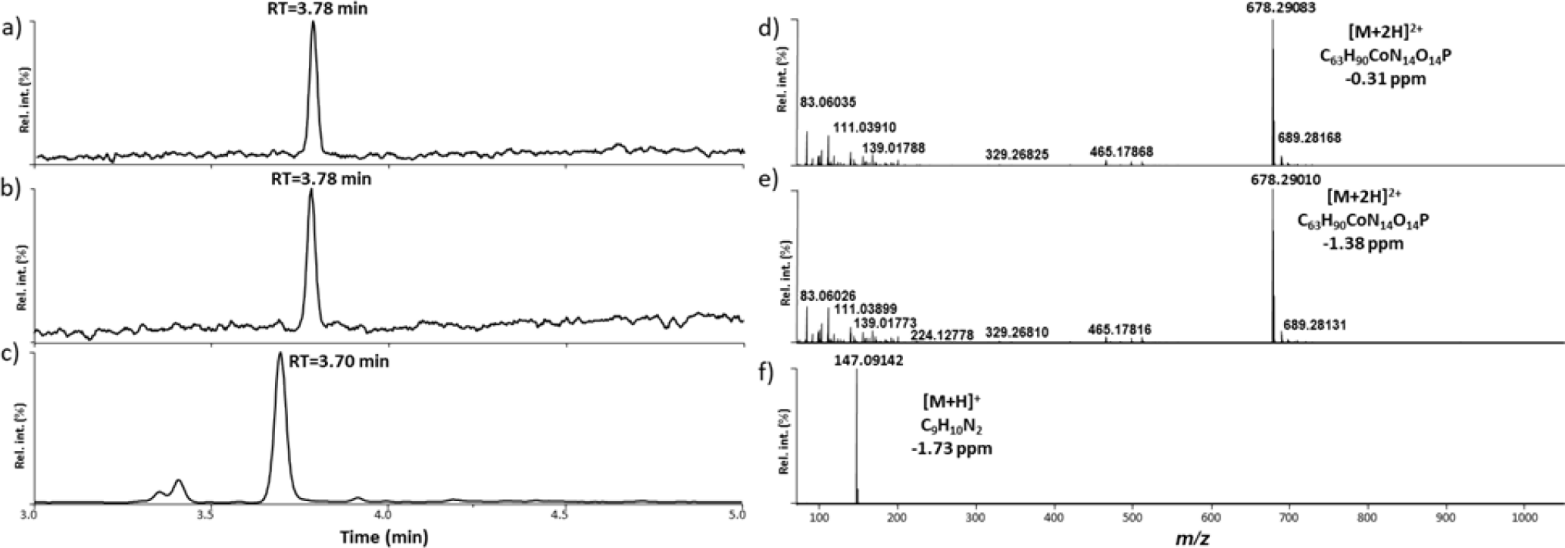
Total ion current measured by liquid chromatography–high-resolution mass spectrometry (LC-HRMS) analysis (positive electrospray ionization (ESI+) full-scan acquisition mode) of (a) a fresh e-liquid sample containing vitamin B12, (b) an e-liquid containing vitamin B12 after the vaping device was used to produce an aerosol and (c) trapped aerosol extract sample produced from the vitamin B12-supplemented vaping device (glass fiber filter pad plus impinger), and (d–f) their subtracted full-scan ESI+ mass spectra at the retention times of 3.78, 3.78, and 3.70 min, respectively. rel int.: relative intensity.

The LC–HRMS analysis of e-liquid from the vaping device confirmed the presence of vitamin B12 at the reported concentration level mentioned by the supplier. Under optimized chromatographic conditions used to analyze this e-liquid, the peak corresponding to vitamin B12 appeared at a retention time (RT) of 3.78 min (Figure 4a–b), which was confirmed by the analysis of the commercially available reference compounds. The doubly charged species at *m*/*z* 678.29083 ([M + 2H]^2+^) indicated the presence of vitamin B12 in the liquid (Figure 4d). Further, the vitamin B12 quantification results of the e-liquid sample matched the expected concentrations listed in the product information. These contradictory results between the e-liquid and exhaled breath measurements prompted us to perform additional quantitative analyses of the vitamin B12-containing e-liquid after vaping device use (i.e., once the e-liquid in the device had reduced in quantity) (Figure 4b,e). Comparing the data from the fresh e-liquid to those from the e-liquid remaining, after extensive product use, revealed a stable vitamin B12 concentration (within the standard variation of analytical measurements), confirming that the vitamin B12 contained in the e-liquid was consumed during the aerosolization process.

We then generated an aerosol using a PSP pump (Figure 1b), as explained in the Experimental section. Aerosols produced from the vitamin B12-supplemented vaping device were trapped on a glass fiber filter pad connected to an impinger by accumulation of 50 artificial exhalations. Compounds trapped on the glass fiber filter pad were extracted in 5 mL of water and mixed with the impinger solution (previously filled with 5 mL of water). The combined extracted fractions were then analyzed by LC–HRMS in the full-scan positive ESI acquisition mode using the same analytical conditions as those used for quantifying vitamin B12 (details in Supporting Information, LC-HRMS conditions, vitamin B12). Analysis of the trapped aerosol extract (glass fiber filter pad plus impinger) revealed a new peak at the RT of 3.70 min, distinct from that of vitamin B12 (RT = 3.78 min) (Figure 4c).

**Figure 5:**
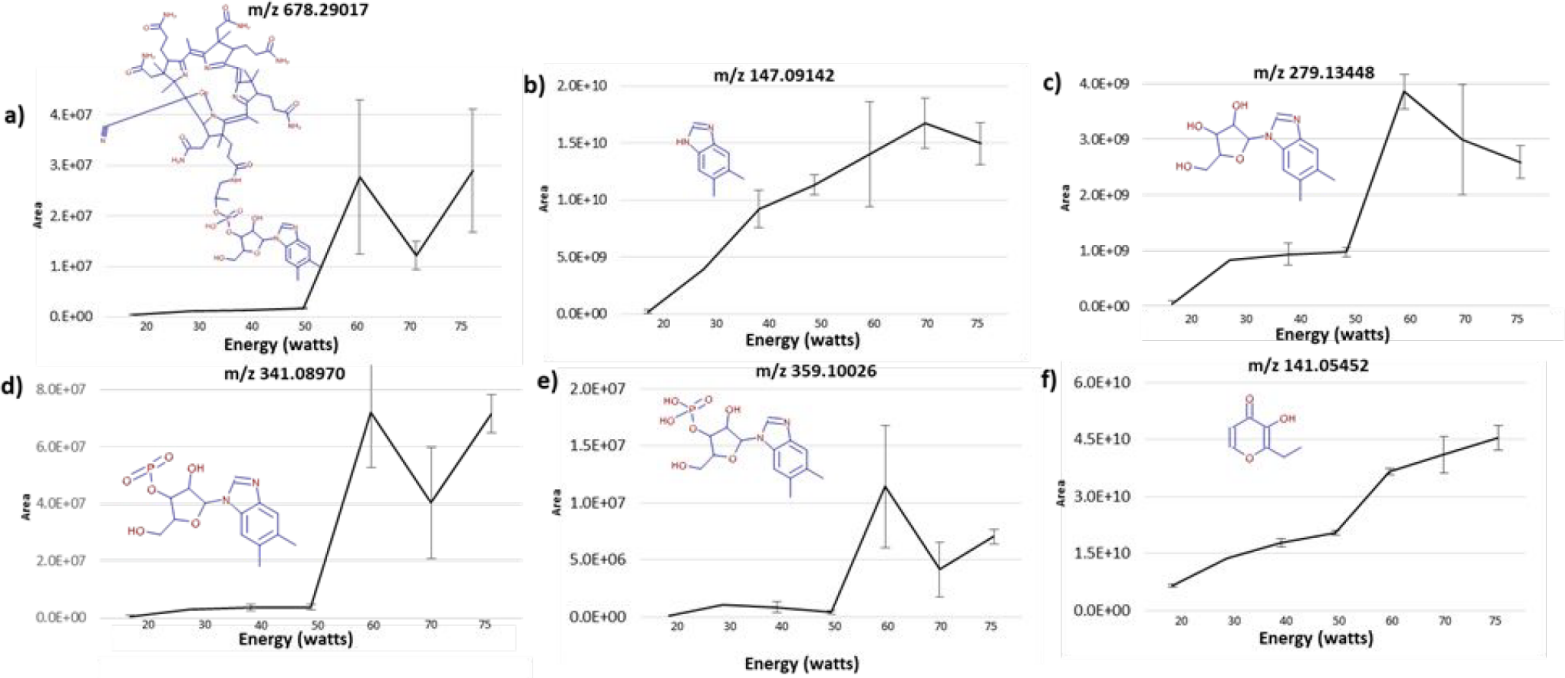
Area response according to the energy profile applied in the in-house thermal aerosol generator filled with a vitamin B12 e-liquid. Each experiment was performed in duplicate from 10 accumulated artificial exhalations. (a) *m*/*z* 678.29017 ([M + 2H]^2+^, −1.19 ppm), (b) *m*/*z* 147.09142 (5,6-dimethylbenzimidazole, −1.73 ppm), (c) *m*/*z* 279.13448 (1.96 ppm), (d) *m*/*z* 341.08970 (0.002 ppm), (e) *m*/*z* 359.10026 (0.10 ppm), and (f) *m*/*z* 141.05452 (ethyl maltol, −0.71 ppm), an ion putatively identified as an added flavor. Chemical structures of the vitamin B12-associated ions and ethyl maltol are depicted in the insets.

The subtracted full-scan ESI mass spectrum showed an intense protonated ion at *m*/*z* 147.09142, which is characteristic of C_9_H_11_N_2_ (−1.73 ppm) (Figure 4f). This mass loss was identified and confirmed to originate from 5,6-dimethylbenzimidazole (C_9_H_10_N_2_, structure reported in Figure 5b) based on the results of the reference standard measured under identical analytical LC–HRMS conditions. 5,6-Dimethylbenzimidazole is a breakdown product of vitamin B12. The coordination of the central cobalt ion in the vitamin B12 molecule with the nitrogen atom of 5,6-dimethylbenzimidazole is pH dependent.^29^ Hence, the e-liquid contained intact vitamin B12 molecules as expected, confirming that the appearance of the breakdown product in the aerosol was not related to the pH of the e-liquid. The exhalation collected after inhalation of aerosol produced from vitamin B12-supplemented vaping device also contained the protonated species *m*/*z* 147.09152, corresponding to C_9_H_11_N_2_ (−1.05 ppm) (Supporting Information, Figure S2). Hypothesizing that the power applied to aerosolize the compounds was extremely high and potentially caused the appearance of breakdown products in the aerosol, we decided to introduce an e-liquid sample from the commercially available vitamin B12-supplemented vaping device into an in-house aerosol generator that can operate by applying different power wattages (20, 30, 40, 50, 60, 70, and 75 W).

The generated aerosols were accumulated from 10 artificial exhalations trapped on a glass fiber filter pad connected in series with a microimpinger containing 5 mL of water. The trapped chemicals were extracted (from the glass fiber filter pad plus microimpinger) with 5 mL of water and analyzed by LC–HRMS to determine the critical energy levels required to produce vitamin B12 degradation products. Figure 5 shows the appearance of the extracted ions from several degradation products originating from vitamin B12, as well as other chemicals present in the e-liquid as flavor constituents (e.g., *m*/*z* 141.05452 ethyl maltol). Figure 5b shows 5,6-dimethylbenzimidazole, and Figure 5c–e shows three other degradation products of vitamin B12. Their concentrations increased at higher energy levels, whereas the intact form of vitamin B12 ([M + H]^+^, C_63_H_89_CoN_14_O_14_P) was found at lower intensity levels (3 × 10^7^ versus 1.5 × 10^10^ ion counts for 5,6-dimethylbenzimidazole). These two compounds were quantified by LC–HRMS, and the data confirmed that the aerosol contained low levels of vitamin B12 (0.044 μg/mL in the trapped aerosol generated at 40 W) relative to 5,6-dimethylbenzimidazole (37.513 μg/mL in the trapped aerosol generated at 40 W) (Supporting Information, Table S2). In comparison, compounds associated with flavor (e.g., ethyl maltol, *m*/*z* 141.05452) exhibited improved aerosolization with increasing energy levels (Figure 5f). The presence of vitamin B12 was confirmed by LC-HRMS but at a very low concentration level (below 1% transfer rate from liquid to aerosol). From these results, we can confirm that vitamin B12 was present at the reported concentration levels in the product, but it was degraded during the aerosolization (this was not associated with the ionization process during the analyses of SESI-HR-MS). These results confirm that aerosol generation conditions are important for successful compound aerosolization. Vitamin B12 has previously been successfully aerosolized using a nebulizer system,^28^ but the aerosolization mechanism differs from that of vaping devices. Nebulizers generate aerosols by applying a dispersing force on the liquid containing the compound of interest,^30^ which can be considered as mechanical aerosolization. In contrast, vaping devices generate an aerosol by heating the liquid containing the compound(s) of interest (i.e., thermal aerosolization).^31^

### Optimization of the Analytical System to Monitor API-Containing Aerosols

The SESI interface shows great potential for monitoring the successful aerosolization of compounds through exhaled breath analysis. To widen the spectrum of the test compounds and ensure proper aerosolization of the compounds from a liquid solution, we developed a system that can deliver aerosols to the SESI interface as an alternative to human exhalation. For this purpose, we connected a PSP, which can operate under defined conditions of aerosol volume, aerosol generation duration, and interval between generated aerosols, to the SESI–HRMS interface (Figure 1b). This approach allowed real-time monitoring of the compounds present in the generated aerosols (artificial exhalation).

To assess this coupling system, we used a melatonin-supplemented vaping device to evaluate the reproducibility of the generated aerosols (*n* = 15) in the SESI-positive ionization mode. As a control experiment, three blank aerosols were analyzed without the device being connected to the PSP (Supporting Information, Figure S3). A total run time of 10 min produced 18 aerosols (3 blanks followed by 15 aerosols from the vaping device). The selected ion extraction of *m*/*z* 233.12845 (mass tolerance of 5 ppm) corresponding to the theoretical protonated species of melatonin confirmed the absence of signals in the first three blank aerosols, whereas the spectra of the next 15 aerosols revealed the presence of melatonin when the device was connected to the SESI–HRMS interface. The coefficient of variation of the peak areas from each aerosol (*n* = 8–18 artificial exhalations) was determined to be 17%, as opposed to the 28% calculated when all aerosol production cycles of the melatonin vaping device were considered. Based on these results, we hypothesized that the device does not properly deliver melatonin during the first five aerosol generations. This result is significant because it demonstrates that the potential application of a developed setup is an important consideration of reproducible API delivery through aerosolization.^32,33^

As an alternative to the previously tested commercial vaping devices, we developed an in-house formulation containing various APIs. The antiviral drug HCQ was solubilized in an appropriate carrier solvent (concentration, 5 mg/mL) and aerosolized using an in-house aerosol generator. In these experiments, the PSP pump was connected to the SESI–HRMS for 5 s, and a 20 mL aerosol volume was produced in a 5-s aerosol generation regimen every 30 s (including the subsequent discharge from the pump during piston downstroke).

**Figure 6:**
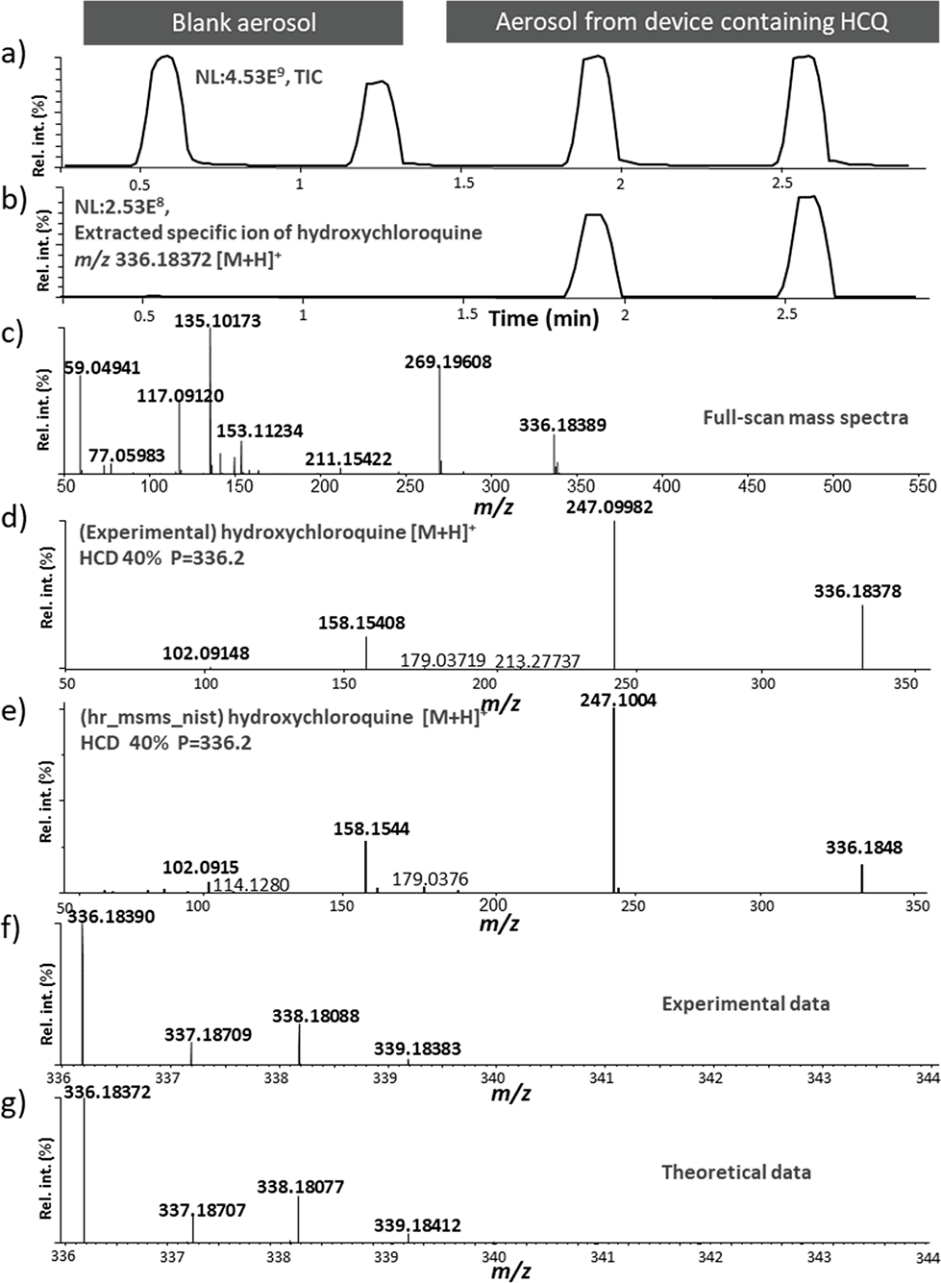
(a) Total ion current and (b) the selected ion extraction of *m*/*z* 336.18372 corresponding to the theoretical protonated species of hydroxychloroquine (HCQ) monitored in the positive secondary electrospray ionization (SESI) mode. Two blank aerosols produced by the programmable syringe pump (PSP) with room air only were monitored first, followed by two aerosols produced by thermal aerosolization of the HCQ solution. (c) Subtracted full-scan SESI mass spectrum in the positive ionization mode (*m*/*z* 50–550) of an aerosol generated with a device containing HCQ. (d) High-energy collision dissociation (HCD) tandem MS spectrum from selecting *m*/*z* 336.2 at HCD 40 (SESI+). (e) HCD tandem MS spectrum of HCQ from the NIST20 library. (f) Full-scan SESI+ spectrum of the protonated species of HCQ (zoomed in view) and (g) the theoretical chlorine isotopic pattern of protonated C_18_H_26_ClN_3_O. rel int.: relative intensities.

Figure 6 depicts a typical profile of the data generated using the developed setup. Overall, a 3 min run time allowed us to monitor the two blank aerosols (only room air) produced by the PSP, followed by the two aerosols produced using the device containing the HCQ solution (Figure 6a). The full-scan SESI+ mass measurements confirmed aerosolization of the API along with possible degradation products and/or impurities that might have been generated through the thermal aerosolization process. Figure 6b shows the selected ion extraction of *m*/*z* 336.18372 corresponding to the theoretical protonated species of HCQ monitored in the SESI+ mode. The SESI+ full-scan spectrum showed a few ions at *m*/*z* 336.18389 corresponding to protonated HCQ (0.52 ppm), and *m*/*z* 59.04941, *m*/*z* 77.05983, *m*/*z* 117.09120, *m*/*z* 135.10173, *m*/*z* 153.11234, *m*/*z* 211.15422, and *m*/*z* 269.19608 corresponding to the propylene glycol species [M-H_2_O + H]^+^ (C_3_H_7_O, 4.55 ppm), [M + H]^+^ (C_3_H_9_O_2_, 1.61 ppm), [2M-2H_2_O + H]^+^ (C_6_H_13_O_2_, 1.66 ppm), [2M-H_2_O + H]^+^ (C_6_H_15_O_3_, 1.18 ppm), [2M + H]^+^ (C_6_H_17_O_4_, 1.34 ppm), [3M-H_2_O + H]^+^ (C_9_H_23_O_5_, 0.99 ppm), and [4M-H_2_O + H]^+^ (C_12_H_28_O_6_, 0.80 ppm), respectively (Figure 6c).

This example highlights that performed real-time measurements showed the presence of several ions in the form of adduct ions for one molecule (e.g., protonated and deprotonated), dimers (when a relatively high concentration of the molecule was present in the sample, up to tetramers for propylene glycol), and ions originating from in-source fragmentation (loss of water or ammonia).

A weakness of real-time analysis (i.e., omitting chromatographic separation) is compound identification when multiple ions are simultaneously detected. To confirm HCQ aerosolization, we generated an HCD tandem MS spectrum for the *m*/*z* 336.2 corresponding to a protonated species of HCQ (Figure 6d) and found a well-matched tandem MS spectrum within the NIST20 library (Figure 6e). Moreover, the Orbitrap revealed an isotopic pattern in the SESI full-scan mode owing to the presence of a chlorine atom in HCQ (Figure 6f–g).

The various optimization processes with this coupling system emphasized the importance of minimizing contamination of the MS detector. We addressed this issue by (a) introducing a nitrogen stream to convey the aerosol flow through the mass detector and (b) manually connecting the test device to the PSP pump–SESI inlet for only 5 s, while the generated aerosol was being delivered, allowing the chemicals to reach the ionization chamber and MS detector. Under these conditions, the total ion current (TIC) showed a clear signal increase with less carryover (back to baseline between artificial exhalations). However, some compounds were retained in the system to a greater extent, and a longer time was required to clean the connections. To avoid compound retention in the system, we decreased the temperature of the sample line to room temperature to reduce compound evaporation from the walls of the tube and increased the temperature of the ionization chamber to improve compound ionization efficiency.

To assess the capability of the system to monitor chemicals of various molecular masses and boiling points in both positive and negative SESI acquisition modes, we tested several compounds under these conditions, including CQ, cannabidiol, and azithromycin. Two drugs were confirmed to be present in the produced aerosol samples, with the corresponding protonated or deprotonated species found for CQ (C_18_H_26_ClN_3_, *m*/*z* 320.18892 as [M + H]^+^ and 318.17455 as [M – H]^−^) and cannabidiol (C_21_H_30_O_2_, *m*/*z* 315.23163 as [M + H]^+^ and 313.21735 [M – H]^−^) (Supporting Information, Figures S4–5)). In contrast, azithromycin (C_38_H_72_N_2_O_12_) was not confirmed in the aerosol in either positive or negative SESI acquisition mode as a singly or doubly charged species (Supporting Information, Figure 6S).

LC–HRMS analyses of the e-liquid confirmed the presence of the azithromycin in the positive ESI acquisition mode, both as singly and doubly charged species, at *m*/*z* 749.51580 and 375.26154, respectively, at a retention time of 5.0 min (Supporting Information Figure S7). No additional peaks corresponding to potential degradation products were observed. These results correlated well with our findings with the vitamin B12 vaping device and the absence of the compound in the generated aerosol sample. The latter results for azithromycin and vitamin B12 indicate that the optimal mass region for acquisition using Super SESI should be below *m*/*z* 600, as previously demonstrated.^34^

Table 1 summarizes all of the compounds monitored in this study and provides additional information on the boiling points and enthalpies of vaporization predicted by Percepta ACD/Laboratories software.^35^ This is particularly significant in aerosol generation devices in which the liquid is immediately heated, causing flash boiling evaporation instead of slow compound-selective distillation. The boiling point of a mixture can be easily decreased by adding water. Further, a volumetric evaporation process, instead of a surface-driven process, enables the phase change to occur at temperatures well below the boiling point of the targeted API.

**Table 1:** List of Monitored Compounds with Some Physicochemical Properties

compound	formula	monoisotopic mass	boiling point (Predicted ACD/Laboratories) (°C at 760 mmHg)	enthalpy of vaporization (kJ/mol)
caffeine	C_8_H_10_N_4_O_2_	194.079827	416.8 ± 37.0	67.0 ± 3.0
chloroquine	C_18_H_26_ClN_3_	319.180977	460.6 ± 40.0	72.1 ± 3.0
cannabidiol	C_21_H_30_O_2_	314.224032	463.9 ± 45.0	75.3 ± 3.0
melatonin	C_13_H_16_N_2_O_2_	232.120629	512.8 ± 40.0	78.4 ± 3.0
hydroxychloroquine	C_18_H_26_ClN_3_O	335.176989	516.7 ± 50.0	83.0 ± 3.0
azithromycin	C_38_H_72_N_2_O_12_	748.507977	822.1 ± 65.0	136.0 ± 6.0
vitamin B12	C_63_H_88_CoN_14_O_14_P	1354.566856	n/a	n/a

## Conclusion

This study demonstrated the ability of Super SESI technology interfaced with an Orbitrap–MS system to perform real-time untargeted analysis of generated aerosols. To monitor compound aerosolization, we modified the commercial technology by connecting a PSP pump and nitrogen stream to the SESI sample line, thereby enabling the flow of thermally generated aerosols to the ionization chamber. The generated aerosol can be chemically analyzed in both positive and negative SESI ionization modes. This soft ionization mode permits intact molecules to represent the main ion signal. High-resolution accurate mass precision of full-scan acquisition and fragmentation pattern of tandem MS experiments allowed us to confirm appropriate drug aerosolization and putatively identify possible drug degradation products.

As a proof of concept, we analyzed several thermally generated aerosols in real time; caffeine, melatonin, HCQ, CQ, and cannabidiol were successfully detected, while vitamin B12 and azithromycin were not (even if all analytes were present in the e-liquids at the reported concentration by the suppliers). Owing to the high-resolution accurate mass measurements, it was possible to interpret the ion adducts, dimers, and in-source fragmentation ions.

We demonstrated the proper aerosolization of compounds at temperatures considerably lower than their predicted boiling points. Consequently, investigations of such formulations are of scientific interest given their practical value in drug delivery processes in terms of decreasing the risks of thermal degradation and production of unwanted toxic compounds. However, it must also be noted that mixtures composed of compounds with significantly distinct properties are certainly more prone to heterogeneous behavior including an increased risk of liquid stratification, which might induce variability in the delivered dose.

The SESI interface and our adapted setup for real-time monitoring of compounds show considerable potential for rapidly assessing aerosolization. Screening different devices and/or investigating the best carrier solvents for product formulations are key factors for improving the transfer of chemicals from the liquid to gas phase. This technique can be easily applied in high-throughput analyses to identify optimal conditions for successful compound aerosolization.

## Supporting Information Available

Table S1. Theoretical and experimental concentrations of caffeine, melatonin, and vitamin B12 in the commercially available e-liquids. Figures S1–S6. Real-time analyses of exhaled breath and aerosols produced by vaping devices containing vitamin B12, chloroquine, cannabidiol, and azithromycin. Figure S7. Liquid chromatography–high-resolution mass spectrometry analysis of an e-liquid containing azithromycin. LC–HRMS conditions for analyses of vitamin B12, caffeine, melatonin, azithromycin, hydroxychloroquine, and chloroquine (PDF)

## References

[ref1] Lavorini F.; Buttini F.; Usmani O. S. 100 Years of Drug Delivery to the Lungs. Handb Exp Pharmacol 2019, 260, 143–159. 10.1007/164_2019_335. 31792683

[ref2] Rubin B. K.; Williams R. W. Emerging aerosol drug delivery strategies: from bench to clinic. Adv. Drug Deliv Rev. 2014, 75, 141–148. 10.1016/j.addr.2014.06.008. 24993613

[ref3] Sanders M. Inhalation therapy: an historical review. Prim Care Respir J. 2007, 16 (2), 71–81. 10.3132/pcrj.2007.00017. 17356785 PMC6634187

[ref4] Strong P.; Ito K.; Murray J.; Rapeport G. Current approaches to the discovery of novel inhaled medicines. Drug Discov Today 2018, 23 (10), 1705–1717. 10.1016/j.drudis.2018.05.017. 29775668

[ref5] Pirozynski M.; Sosnowski T. R. Inhalation devices: from basic science to practical use, innovative vs generic products. Expert Opin Drug Deliv 2016, 13 (11), 1559–1571. 10.1080/17425247.2016.1198774. 27267298

[ref6] Pasqua E.; Hamblin N.; Edwards C.; Baker-Glenn C.; Hurley C. Developing inhaled drugs for respiratory diseases: A medicinal chemistry perspective. Drug Discov Today 2022, 27 (1), 134–150. 10.1016/j.drudis.2021.09.005. 34547449

[ref7] Basanez T.; Majmundar A.; Cruz T. B.; Allem J. P.; Unger J. B. E-cigarettes Are Being Marketed as ″Vitamin Delivery″ Devices. Am. J. Public Health 2019, 109 (2), 194–196. 10.2105/AJPH.2018.304804. 30649935 PMC6336045

[ref8] Cowan E. A.; Tran H.; Gray N.; Perez J. J.; Watson C.; Blount B. C.; Valentin-Blasini L. A gas chromatography-mass spectrometry method for quantifying squalane and squalene in aerosol emissions of electronic cigarette, or vaping products. Talanta 2022, 238, 122985 10.1016/j.talanta.2021.122985. 34857320

[ref9] Bentley M. C.; Almstetter M.; Arndt D.; Knorr A.; Martin E.; Pospisil P.; Maeder S. Comprehensive chemical characterization of the aerosol generated by a heated tobacco product by untargeted screening. Anal Bioanal Chem. 2020, 412 (11), 2675–2685. 10.1007/s00216-020-02502-1. 32072212 PMC7136312

[ref10] Rawlinson C.; Martin S.; Frosina J.; Wright C. Chemical characterisation of aerosols emitted by electronic cigarettes using thermal desorption-gas chromatography-time of flight mass spectrometry. J. Chromatogr A 2017, 1497, 144–154. 10.1016/j.chroma.2017.02.050. 28381359

[ref11] Muller M.; Eichler P.; D’Anna B.; Tan W.; Wisthaler A. Direct Sampling and Analysis of Atmospheric Particulate Organic Matter by Proton-Transfer-Reaction Mass Spectrometry. Anal. Chem. 2017, 89 (20), 10889–10897. 10.1021/acs.analchem.7b02582. 28911223

[ref12] Forbes T. P.; Krauss S. T. Confined DART-MS for Rapid Chemical Analysis of Electronic Cigarette Aerosols and Spiked Drugs. J. Am. Soc. Mass Spectrom. 2021, 32 (8), 2274–2280. 10.1021/jasms.1c00103. 34184882 PMC9969341

[ref13] Bacsik Z.; McGregor J.; Mink J. FTIR analysis of gaseous compounds in the mainstream smoke of regular and light cigarettes. Food Chem. Toxicol. 2007, 45 (2), 266–271. 10.1016/j.fct.2006.08.018. 17046136

[ref14] Smith D.; Spaněl P. Ambient analysis of trace compounds in gaseous media by SIFT-MS. Analyst 2011, 136 (10), 2009–2032. 10.1039/c1an15082k. 21431189

[ref15] Breiev K.; Burseg K. M.; O’Connell G.; Hartungen E.; Biel S. S.; Cahours X.; Colard S.; Mark T. D.; Sulzer P. An online method for the analysis of volatile organic compounds in electronic cigarette aerosol based on proton transfer reaction mass spectrometry. Rapid Commun. Mass Spectrom. 2016, 30 (6), 691–697. 10.1002/rcm.7487. 26864521 PMC4755144

[ref16] Frege C.; Asgari M.; Steiner S.; Ferreira S.; Majeed S.; Lucci F.; Frentzel S.; Hoeng J.; Kuczaj A. K. Assessment of Single-Photon Ionization Mass Spectrometry for Online Monitoring of in Vitro Aerosol Exposure Experiments. Chem. Res. Toxicol. 2020, 33 (2), 505–514. 10.1021/acs.chemrestox.9b00381. 31909610

[ref17] Zuth C.; Vogel A. L.; Ockenfeld S.; Huesmann R.; Hoffmann T. Ultrahigh-Resolution Mass Spectrometry in Real Time: Atmospheric Pressure Chemical Ionization Orbitrap Mass Spectrometry of Atmospheric Organic Aerosol. Anal. Chem. 2018, 90 (15), 8816–8823. 10.1021/acs.analchem.8b00671. 29961316

[ref18] Lee C. P.; Riva M.; Wang D.; Tomaz S.; Li D.; Perrier S.; Slowik J. G.; Bourgain F.; Schmale J.; Prevot A. S. H.; Baltensperger U.; George C.; El Haddad I. Online Aerosol Chemical Characterization by Extractive Electrospray Ionization-Ultrahigh-Resolution Mass Spectrometry (EESI-Orbitrap). Environ. Sci. Technol. 2020, 54 (7), 3871–3880. 10.1021/acs.est.9b07090. 32146813

[ref19] Lan J.; Gisler A.; Bruderer T.; Sinues P.; Zenobi R. Monitoring peppermint washout in the breath metabolome by secondary electrospray ionization-high resolution mass spectrometry. J. Breath Res. 2021, 15 (2), 026003 10.1088/1752-7163/ab9f8a. 32575094

[ref20] Singh K. D.; Tancev G.; Decrue F.; Usemann J.; Appenzeller R.; Barreiro P.; Jauma G.; Macia Santiago M.; Vidal de Miguel G.; Frey U.; Sinues P. Standardization procedures for real-time breath analysis by secondary electrospray ionization high-resolution mass spectrometry. Anal Bioanal Chem. 2019, 411 (19), 4883–4898. 10.1007/s00216-019-01764-8. 30989265 PMC6611759

[ref21] Tejero Rioseras A.; Singh K. D.; Nowak N.; Gaugg M. T.; Bruderer T.; Zenobi R.; Sinues P. M. Real-Time Monitoring of Tricarboxylic Acid Metabolites in Exhaled Breath. Anal. Chem. 2018, 90 (11), 6453–6460. 10.1021/acs.analchem.7b04600. 29767961

[ref22] Garcia-Gomez D.; Gaisl T.; Barrios-Collado C.; Vidal-de-Miguel G.; Kohler M.; Zenobi R. Real-Time Chemical Analysis of E-Cigarette Aerosols By Means Of Secondary Electrospray Ionization Mass Spectrometry. Chemistry 2016, 22 (7), 2452–2457. 10.1002/chem.201504450. 26773448

[ref23] Ueno H.; Takahashi Y.; Suemitsu S.; Murakami S.; Kitamura N.; Wani K.; Matsumoto Y.; Okamoto M.; Ishihara T. Caffeine inhalation effects on locomotor activity in mice. Drug Dev. Ind. Pharm. 2020, 46 (5), 788–794. 10.1080/03639045.2020.1753064. 32292092

[ref24] Yuan Z. C.; Li W.; Wu L.; Huang D.; Wu M.; Hu B. Solid-Phase Microextraction Fiber in Face Mask for in Vivo Sampling and Direct Mass Spectrometry Analysis of Exhaled Breath Aerosol. Anal. Chem. 2020, 92 (17), 11543–11547. 10.1021/acs.analchem.0c02118. 32786499

[ref25] Campos F. L.; da Silva-Junior F. P.; de Bruin V. M.; de Bruin P. F. Melatonin improves sleep in asthma: a randomized, double-blind, placebo-controlled study. Am. J. Respir Crit Care Med. 2004, 170 (9), 947–951. 10.1164/rccm.200404-488OC. 15306531

[ref26] Nunes D.M.; Mota R.M.S.; Machado M.O.; Pereira E.D.B.; de Bruin V.M.S.; de Bruin P.F.C. Effect of melatonin administration on subjective sleep quality in chronic obstructive pulmonary disease. Braz. J. Med. Biol. Res. 2008, 41 (10), 926–931. 10.1590/S0100-879X2008001000016. 19030713

[ref27] Reiter R. J.; Tan D. X.; Galano A. Melatonin: exceeding expectations. Physiology (Bethesda) 2014, 29 (5), 325–333. 10.1152/physiol.00011.2014. 25180262

[ref28] Smith F. J.; Monto R. W.; Rebuck J. W. B12 inhalation therapy in pernicious anemia. Trans. Am. Clin. Climatol. Assoc. 1952, 64, 27–39. 13136209 PMC2248818

[ref29] oProinsias K.; Giedyk M.; Gryko D. Vitamin B12: chemical modifications. Chem. Soc. Rev. 2013, 42 (16), 6605–6619. 10.1039/c3cs60062a. 23715409

[ref30] Dhanani J.; Fraser J. F.; Chan H. K.; Rello J.; Cohen J.; Roberts J. A. Fundamentals of aerosol therapy in critical care. Crit Care 2016, 20 (1), 269 10.1186/s13054-016-1448-5. 27716346 PMC5054555

[ref31] Brown C. J.; Cheng J. M. Electronic cigarettes: product characterisation and design considerations. Tob Control 2014, 23 (Suppl 2), ii4–10. 10.1136/tobaccocontrol-2013-051476. 24732162 PMC3995271

[ref32] Byron P. R. Drug delivery devices: issues in drug development. Proc. Am. Thorac Soc. 2004, 1 (4), 321–328. 10.1513/pats.200403-023MS. 16113453

[ref33] Dhand R. Reproducible Dosing With a Jet Nebulizer During Invasive Mechanical Ventilation. Respir Care 2020, 65 (8), 1223–1224. 10.4187/respcare.08279. 32712586

[ref34] Bruderer T.; Gaugg M. T.; Cappellin L.; Lopez-Hilfiker F.; Hutterli M.; Perkins N.; Zenobi R.; Moeller A. Detection of Volatile Organic Compounds with Secondary Electrospray Ionization and Proton Transfer Reaction High-Resolution Mass Spectrometry: A Feature Comparison. J. Am. Soc. Mass Spectrom. 2020, 31, 1632 10.1021/jasms.0c00059. 32584571

[ref35] https://www.acdlabs.com/products/percepta/ (accessed on February 17, 2022).

