# Supplementary material for: Application of Secondary Electrospray Ionization Coupled with High-Resolution Mass Spectrometry in Chemical Characterization of Thermally Generated Aerosols
Source: J Am Soc Mass Spectrom. 2022 Oct 11;33(11):2147–55. doi: 10.1021/jasms.2c00222 (PMC9634908; doi:10.1021/jasms.2c00222)
Supplement: Supplementary file 1 — js2c00222_si_001.pdf [file js2c00222_si_001.pdf]

Supporting information

Application of Secondary Electrospray Ionization Coupled with High-Resolution Mass Spectrometry in Chemical Characterization of Thermally Generated Aerosols

Tanja Zivkovic Semren*[1], Shoaib Majeed[1], Maria Fatarova[1], Csaba Laszlo[1], Claudius Pak[1], Sandro Steiner[1], Guillermo Vidal-de-Miguel[2], Arkadiusz Kuczaj[1], Anatoly Mazurov[1], Manuel C. Peitsch[1], Nikolai V. Ivanov[1], Julia Hoeng[1], Philippe A. Guy[1]

[1] PMI R&D, Philip Morris Products S.A., Quai Jeanrenaud 5, CH-2000 Neuchatel, Switzerland

[2] Fossil Ion Technology, Promalaga, C. la Gitanilla, 17, 29004 Malaga, Spain

Corresponding author e-mail: tanja.zivkovicsemren@pmi.com

Quantification of caffeine, melatonin, and vitamin B12 in e-liquid from commercially available vaping devices

The specific doses of the compounds in one aerosol generation were listed in the product information. The final concentration of each compound was calculated by considering the available volume of generated aerosol per vapor product and assuming complete aerosolization of compounds during vapor production. The e-liquids were retrieved from the commercially available vaping devices, and the compounds of interest were quantified by liquid chromatography coupled to high-resolution mass spectrometry (LC–HRMS).

Table S1. Theoretical and experimental concentrations of caffeine, melatonin, and vitamin B12 in e-liquid from commercially available vaping devices.

Compound	Theoretical concentration (mg/mL)	Experimental concentration (mg/mL)
Caffeine	7.53	6.39
Melatonin	47.06	45.30
Vitamin B12	6.86	6.08

Real-time analysis of compounds

Vitamin B12

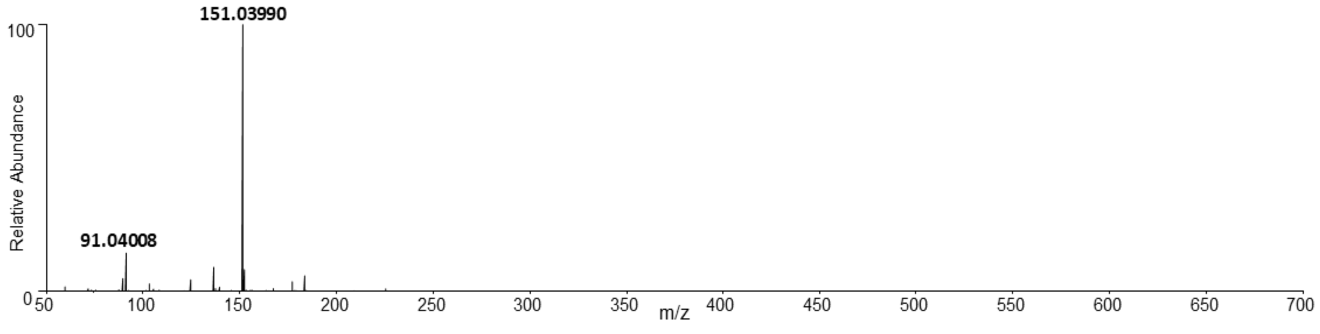

Figure S1. Full-scan negative secondary electrospray mass spectrum (*m/z* 50–700) of exhaled breath after inhalation of aerosol from a vitamin B12-supplemented commercially vaping device analyzed by SESI–HRMS.

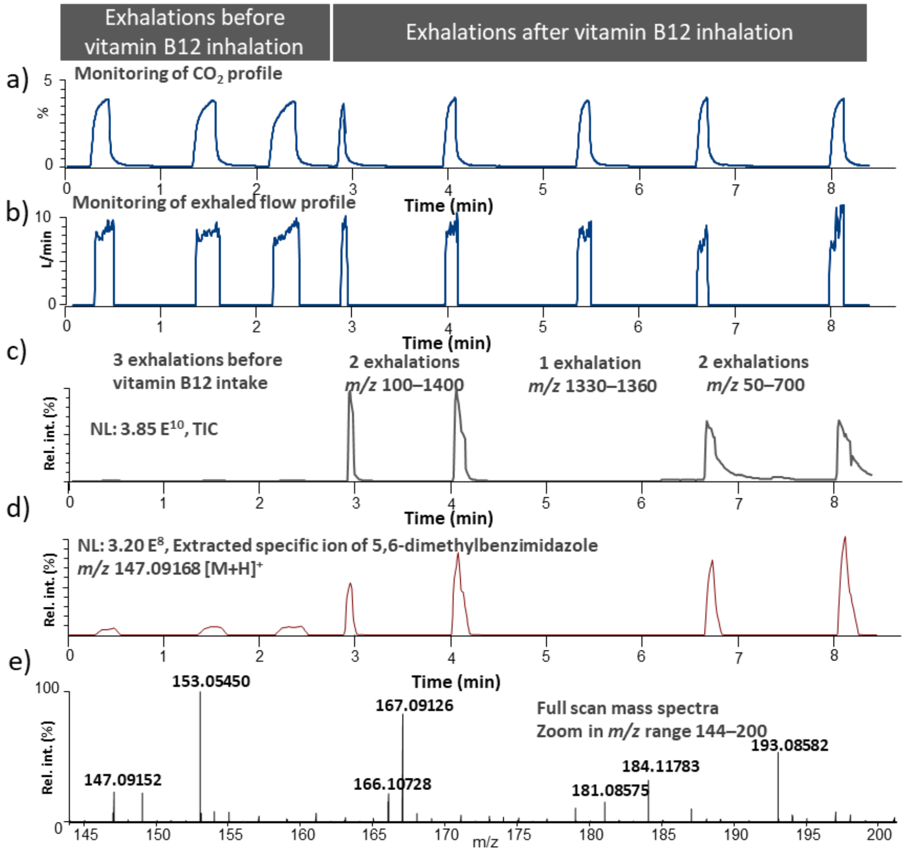

Figure S2. a) CO_2 levels and b) total volume of exhaled breath monitored by the Exhalion software. c) Total ion current measured during the time course of the exhalation experiments, with three exhalations before inhalation of aerosol from a vitamin B12-supplemented commercially vaping device followed by two exhalations measured by scanning m/z 100–1400, one measured by scanning m/z 1330–1360, and the last two measured by scanning m/z 50–700 in the positive secondary electrospray ionization (SESI) mode. d) Selected ion extraction (mass tolerance 5 ppm) of *m/z* 147.09168 corresponding to the theoretical protonated species of 5,6-dimethylbenzimidazole. e) Substracted full-scan positive SESI mass spectrum generated by scanning *m/z* 144–200. Rel. int. – relative intensities.

Table S2. Concentrations of vitamin B12 and 5,6-dimethylbenzimidazole in filter extracts mixed with impinger solution obtained by trapping 10 artificial exhalations generated by thermal aerosolization under different applied powers.

Power (watts)	Vitamin B12 (µg/mL)	5,6-Dimethylbenzimidaz-ole(µg/mL)
20	0.007	0.029
30	0.041	8.310
40	0.044	37.513
50	0.056	50.889
60	0.270	38.363
70	0.117	47.518
75	0.283	41.000

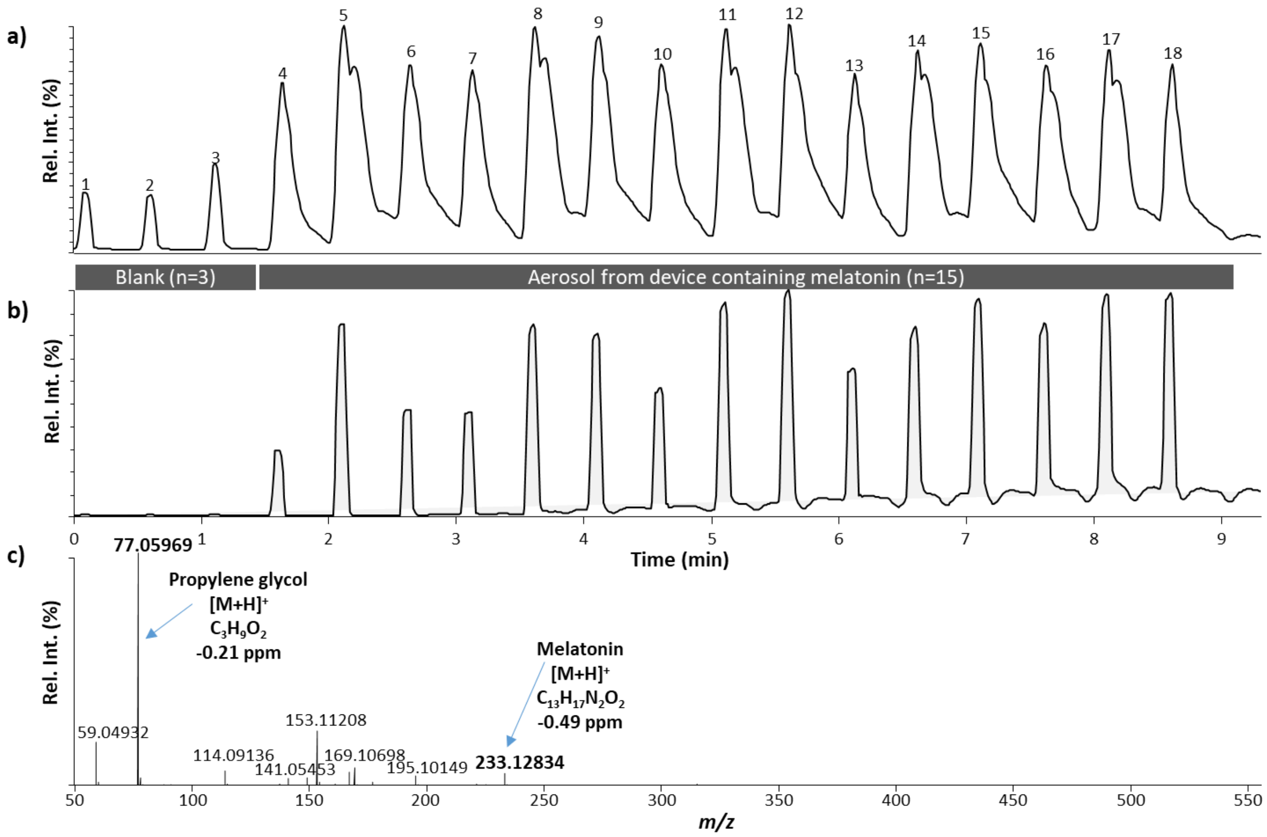

Figure S3. a) Total ion current and b) the corresponding extracted protonated species (*m/z* 233.12845) of melatonin in an e-vapor aerosol monitored in positive electrospray ionization mode. Three blank puffs (the device was not connected to the programmable syringe pump) followed by 15 puffs from the e-vapor product. c) Subtracted full-scan mass spectrum of a characteristic puff from the e-vapor product.

Chloroquine

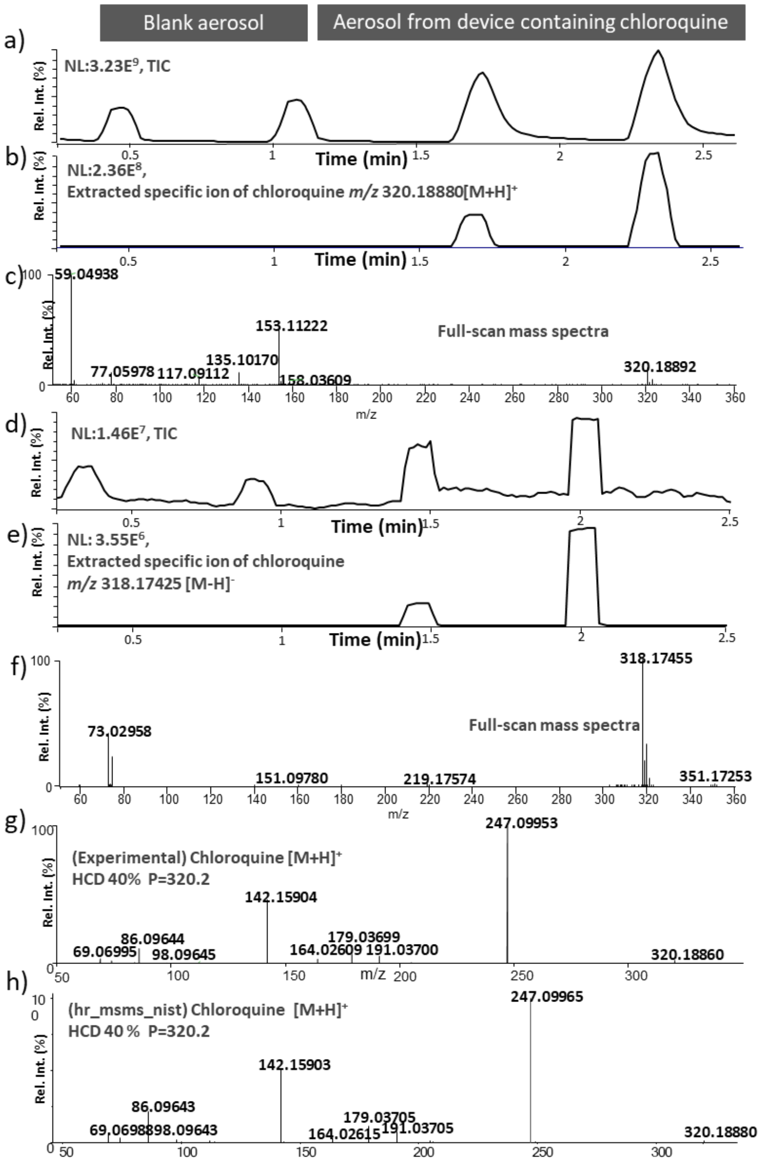

Figure S4. a) Total ion current and **b)** the selected ion extraction of *m/z* 320.18880 corresponding to the theoretical protonated species of chloroquine monitored in positive secondary electrospray ionization (SESI) mode. Two blank aerosols produced by the programmable syringe pump (PSP) with room air only were monitored first, followed by two aerosols produced by thermal aerosolization of the CQ solution. **c)** Subtracted full-scan SESI mass spectrum in the positive ionization mode (m/z 50–360) of an aerosol generated with a device containing CQ. **d)** Total ion current and **e)** the selected ion extraction of *m/z* 318.17425 corresponding to the theoretical deprotonated species of chloroquine monitored in negative SESI mode. Two blank aerosols were monitored first, followed by two aerosols produced by thermal aerosolization of the CQ solution. **f)** Subtracted full-scan SESI mass spectrum in the negative ionization mode (m/z 50–360) of an aerosol generated with a device containing CQ. **g)** High-energy collision dissociation (HCD) tandem MS spectrum of *m/z* 320.2 at HCD 40 (SESI+). **h)** HCD tandem MS spectrum of CQ from the NIST20 library Rel. int. – relative intensities.

Cannabidiol

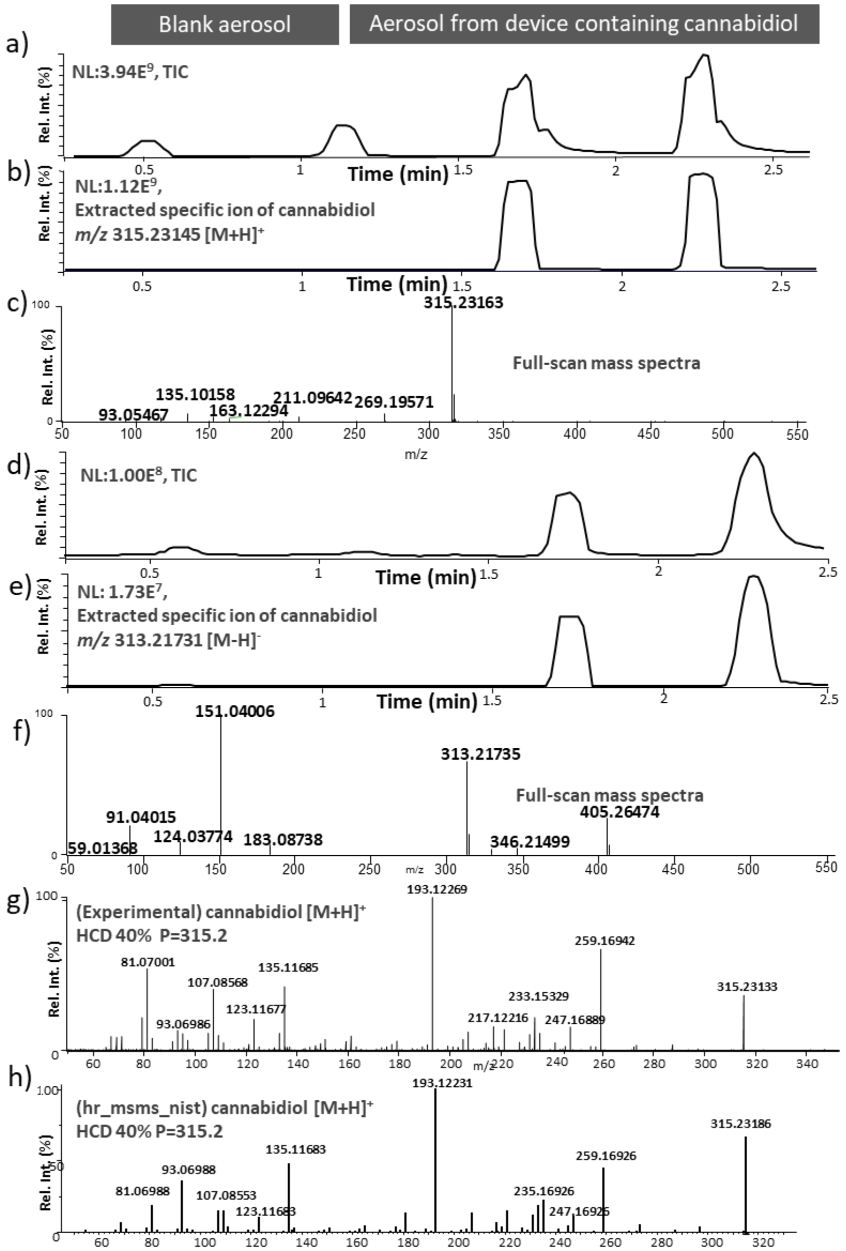

Figure S5. a) Total ion current and **b)** the selected ion extraction of *m/z* 315.23145 corresponding to the theoretical protonated species of cannabidiol monitored in positive secondary electrospray ionization (SESI) mode. Two blank aerosols produced by the programmable syringe pump (PSP) with room air only were monitored first, followed by two aerosols produced by thermal aerosolization of the cannabidiol solution. **c)** Subtracted full-scan SESI mass spectrum in the positive ionization mode (m/z 50–550) of an aerosol generated with a device containing cannabidiol. **d)** Total ion current and **e)** the selected ion extraction of *m/z* 313.21731 corresponding to the theoretical deprotonated species of cannabidiol monitored in negative SESI mode. Two blank aerosols were monitored first, followed by two aerosols produced by thermal aerosolization of the cannabidiol solution. **f)** Subtracted full-scan SESI mass spectrum in the negative ionization mode (m/z 50–550) of an aerosol generated with a device containing cannabidiol. **g)** High-energy collision dissociation (HCD) tandem MS spectrum of *m/z* 315.2 at HCD 40 (SESI+). **h)** HCD tandem MS spectrum of cannabidiol from the NIST20 library Rel. int. – relative intensities.

Azithromycin

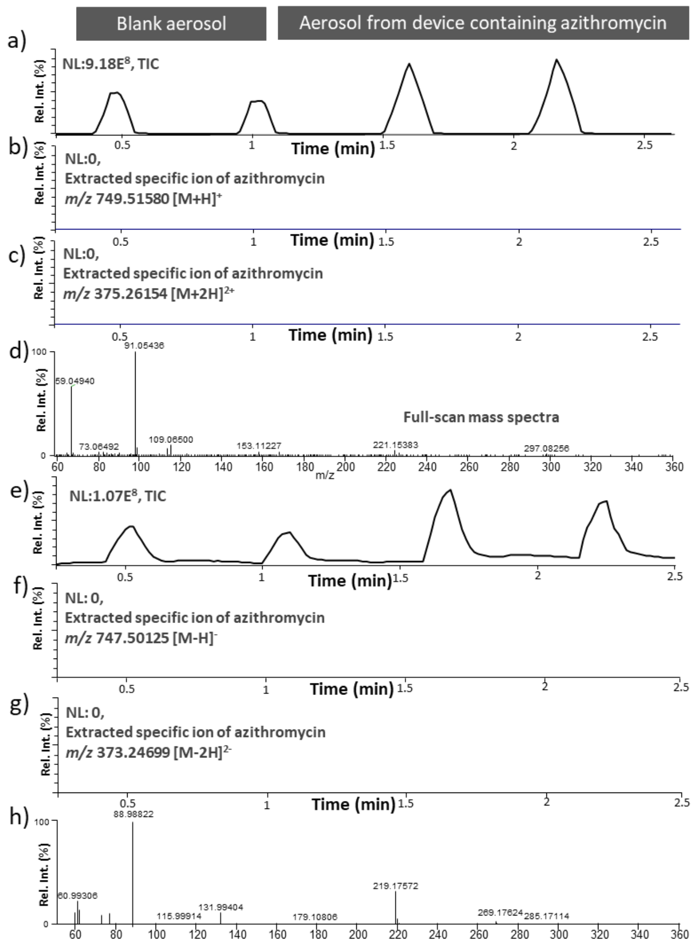

Figure S6. a) Total ion current and selected ion extraction of **b)** *m/z* 749.51580 and **c)** *m/z* 375.26154 corresponding to the theoretical protonated singly and doubly charged species of azithromycin monitored in positive secondary electrospray ionization (SESI) mode. Two blank aerosols produced by the programmable syringe pump (PSP) with room air only were monitored first, followed by two aerosols produced by thermal aerosolization of the azithromycin solution. **d)** Subtracted full-scan SESI mass spectrum in the positive ionization mode (m/z 50–360) of an aerosol generated with a device containing azithromycin. **e)** Total ion current and selected ion extraction of **f)** *m/z* 747.50125 and **g)** *m/z* 373.24699 corresponding to the theoretical deprotonated singly and doubly charged species of azithromycin monitored in negative SESI mode. Two blank aerosols were monitored first, followed by two aerosols produced by thermal aerosolization of the azithromycin solution. **f)** Subtracted full-scan SESI mass spectrum in the negative ionization mode (m/z 50–360) of an aerosol generated with a device containing azithromycin.

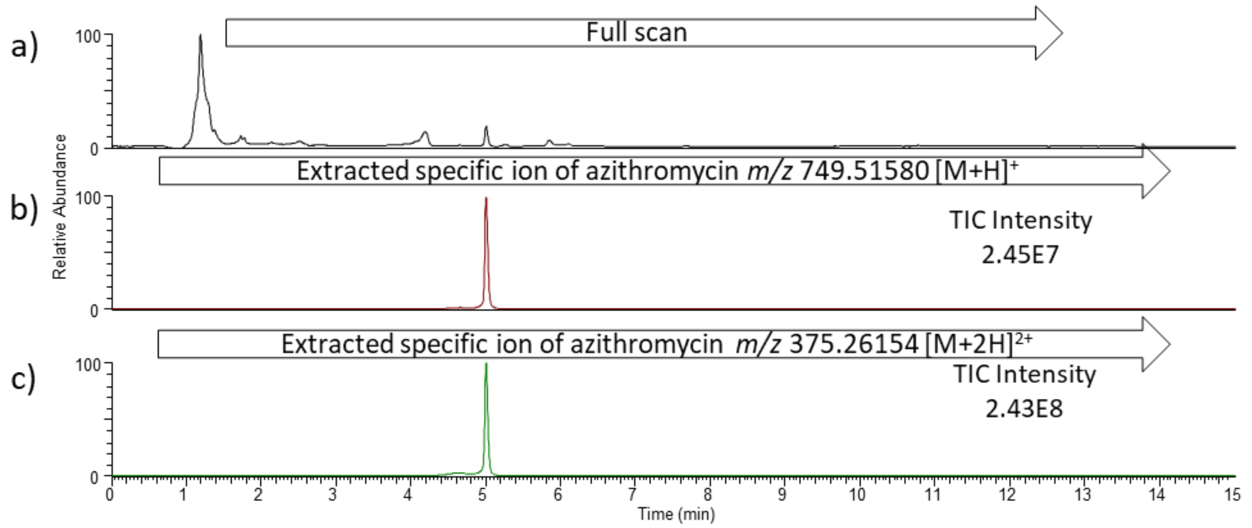

Figure S7. **a)** Total ion current and the corresponding extracted protonated species **b)** *m/z* 749.51580 and **c)** *m/z* 375.26154 in a liquid solution containing azithromycin, analyzed by LC–HRMS in positive electrospray ionization mode.

LC–HRMS conditions

Target compounds in the e-liquids and extracts were quantified by liquid chromatography coupled to high-resolution accurate mass spectrometry (Vanquish Duo–Q Exactive HF system, LC–HRMS; Thermo Fisher Scientific, Waltham, MA, USA). By default, a 5-µL volume was injected into the LC–HRMS system.

Vitamin B12

A Hypersil Gold C18 column (50 x 2.1 mm; 1.9 µm; Thermo Fisher Scientific, Waltham, MA, USA) was used to ensure proper retention of vitamin B12 in the column. The samples were diluted to fit a calibration curve built with six calibrant levels (5–1000 ng/mL). The mobile phase solvents were A) water with 0.1% formic acid and B) acetonitrile with 0.1% formic acid, running at a flow rate of 500 µL/min. The gradient condition was set from 5% B to 95% B in 3 min, staying at 95% B for 2 min before going back to the initial 5% B condition at 5.01 min, with an additional 1 min for column re-equilibration. Full-scan analysis was realized in positive electrospray ionization mode using the following settings: ion spray voltage, 3500 V; capillary and auxiliary temperatures, 256°C and 413°C , respectively; sheath, auxiliary, and sweep gas flow, 48, 11, and 2, respectively; mass resolution, 60,000 (*m/z* 200); mass range, *m/z* 100–1400.

The 5,6-dimethylbenzimidazole reference standard was also analyzed under the same conditions to confirm the identity of vitamin B12 breakdown products. The samples were diluted to fit a calibration curve built from six calibrant levels (5–120 ng/mL).

Caffeine and melatonin

A Hypersil Gold C18 column (15 x 2.1 mm; 1.9 µm; Thermo Fisher Scientific, Waltham, MA, USA) was used to ensure proper retention of caffeine and melatonin on the column. The samples were diluted to fit a calibration curve built from eight calibrant levels (0.005–10 µg/mL). The mobile phase solvents were A) water with 0.1% formic acid and B) acetonitrile with 0.1% formic acid, running at a flow rate of 500 µL/min. The gradient condition was set from 5% B to 95% B in 10 min, staying at 95% B for 2 min before going back to the initial 5% B condition at 12.01 min, with an additional 3 min for column re-equilibration. Full-scan analysis was realized in positive electrospray ionization mode using the following settings: ion spray voltage, 3500 V; capillary and auxiliary temperatures, 280°C and 400°C, respectively; sheath, auxiliary, and sweep gas flow, 50, 15, and 2, respectively; mass resolution, 60,000 (*m/z* 200); mass range, *m/z* 70–1050.

Azithromycin

An Acquity UPLC BEH amide column (15 x 2.1 mm; 1.7 µm; Waters Corp., Milford, MA, USA) was used to ensure proper retention of azithromycin in the column. Azithromycin was not quantified. The mobile phase solvents were A) 10 mM ammonium formate in water with 0.1% formic acid and B) acetonitrile with 0.1% formic acid, running at a flow rate of 500 µL/min. The gradient condition was set from 90% to 50% B in 15 min, staying at 50% A for 3 min before going back to the initial 90% B conditions at 18.1 min, with an additional 2 min for column re-equilibration. Full-scan analysis was realized in positive electrospray ionization mode using the following settings: ion spray voltage, 3500 V; capillary and auxiliary temperatures, 280°C and 400°C, respectively; sheath, auxiliary, and sweep gas flow, 50, 15, and 2, respectively; mass resolution, 60,000 (*m/z* 200); mass range, *m/z* 70–1050.

Hydroxychloroquine and chloroquine

An Acquity UPLC BEH amide column (50 x 2.1 mm; 1.7 µm; Waters Corp., Milford, MA, USA) was used to ensure proper retention of hydroxychloroquine and chloroquine in the column. The samples were diluted to fit a calibration curve built from 10 calibrant

levels (10–250 ng/mL). The mobile phase solvents were A) 10 mM ammonium formate in water with 0.1% formic acid and B) acetonitrile with 0.1% formic acid, running at a flow rate of 500 µL/min. The gradient condition was set from 90% B to 50% B in 3 min, staying at 50% B for 1.4 min before going back to the initial 90% B conditions at 4.5 min, with an additional 1 min for column re-equilibration. Full-scan analysis was realized in positive electrospray ionization mode using the following settings: ion spray voltage, 3500 V; capillary and auxiliary temperatures, 269°C and 438°C, respectively; sheath, auxiliary, and sweep gas flow, 53, 14, and 3, respectively; mass resolution, 30,000 (*m/z* 200); mass range, *m/z* 50–350.
